# An Unclassified Deletion Involving the Proximal Short Arm of Chromosome 10: A New Syndrome?

**DOI:** 10.3390/genes15060650

**Published:** 2024-05-21

**Authors:** Graziano Santoro, Mariarosaria Incoronato, Edoardo Spagnoli, Ilaria Gabbiato, Simona Contini, Marta Piovan, Maurizio Ferrari, Cristina Lapucci, Daniela Zuccarello

**Affiliations:** 1 Genetic Unit, SYNLAB Italia, Via B. L. Pavoni 18, Castenedolo, 25014 Brescia, Italy; edoardo.spagnoli@synlab.it (E.S.); simona.contini@synlab.it (S.C.); marta.piovan@synlab.it (M.P.); cristina.lapucci76@gmail.com (C.L.); 2 IRCCS, SYNLAB SDN, Via Gianturco 113, 80143 Napoli, Italy; mariarosaria.incoronato@synlab.it (M.I.); maurizio.ferrari@synlab.it (M.F.); 3 UOC Genetica Epidemiologia Clinica, Azienda Ospedale Università di Padova, Via Giustiniani 2, 35128 Padova, Italy; ilaria.gabbiato@gmail.com (I.G.); daniela.zuccarello@unipd.it (D.Z.)

**Keywords:** 10p11.2 deletion, array CGH, *WAC* gene

## Abstract

To date, only 13 studies have described patients with large overlapping deletions of 10p11.2-p12. These individuals shared a common phenotype characterized by intellectual disability, developmental delay, distinct facial dysmorphic features, abnormal behaviour, visual impairment, cardiac malformation, and cryptorchidism in males. Molecular cytogenetic analysis revealed that the deletion in this chromosomal region shares a common smallest region of overlap (SRO) of 80 kb, which contains only the *WAC* gene (WW-domain-containing adaptor with coiled coil). In this clinical case report, we report a 5-year-old girl, born from non-consanguineous parents, with a 10p11.22p11.21 microdeletion. She presents clinical features that overlap with other patients described in the literature, such as dysmorphic traits, speech delay, and behavioural abnormalities (hyperactivity), even though the *WAC* gene is not involved in the microdeletion. Our results are the first to highlight that the deletion described here represents a contiguous gene syndrome that is enough to explain the distinct phenotype but partially overlaps with the previous cases reported in the literature, even though the same genes are not involved. In particular, in this study, we speculate about the role of the *WAC* gene that seems to be associated with normal motor development. In fact, we found that our patient is the only one described in the literature with a large deletion in the 10p11.22p11.21 region without the involvement of the *WAC* gene deletion, and, interestingly, the patient did not have motor delay.

## 1. Introduction

Chromosomal abnormalities, involving the short arm of chromosome 10, have been rarely reported. Only 13 patients with large overlapping deletions of 10p11.2-p12 have been described in the literature [1,2,3,4,5,6,7]. These individuals shared a common phenotype characterized by intellectual disability, developmental delay, distinct facial dysmorphic features, abnormal behaviour, visual impairment, cardiac malformation, and cryptorchidism in males. Molecular cytogenetic analysis revealed that the deletion in this chromosomal region shares a common smallest region of overlap (SRO) of 80 kb, which only contains the *WAC* gene (WW-domain-containing adaptor with coiled coil) (OMIM #615049) [5].

Karyotype is the gold standard for analysing the detection of numerical chromosomal abnormalities (aneuploidies) and structural chromosomal rearrangements. At the 500–550 band level, the limit for detecting genomic rearrangements is expected to be above 5–10 Mb, while copy number variation (CNV) analysis is nowadays routinely offered to patients with developmental delay, autistic spectrum disorder, and dysmorphic features because it is useful for the detection of chromosomal rearrangements smaller than 5 Mb. The recurrent CNVs associated with new syndromes are often related to abnormalities in a region that show a variable size and breakpoints with only some small areas of overlap, in which genes that seem to determine the phenotype are shared [8].

Here, we report a female 5-year-old child with a microdeletion in 10p11.22p11.21. Although this patient exhibited dysmorphic traits, speech delay, and behavioural abnormalities (hyperactivity) that overlap with other clinical cases described in the literature [1,2,3,4,5,6,7], the same cannot be said for the case of genetic modification.

## 2. Materials and Methods

### 2.1. Clinical Report

The female patient was the first child born to non-consanguineous healthy parents. The mother and the father were 39 and 56 years old, respectively, at the time of the patient’s birth. She presented severe plagiocephaly, facial asymmetry, prognathism and an open bite, epicanthus, long palpebral fissures, a bulbous nasal tip, cupped ears, a short neck, a bilateral single palmar crease, flat feet, and visual defects (photo not shown). Moreover, she had small-joint laxity, as well as soft and elastic skin. After genetic counselling, the geneticist suspected a chromosomal anomaly and suggested Karyotype analysis. The patient underwent genetic testing after written informed consent was obtained from her parents.

### 2.2. Cytogenetic Analyses

The conventional karyotyping of peripheral blood lymphocytes was performed using Q-band analysis. The result was further validated through array CGH, which accurately detected the breakpoints of the deletion, the genomic size, and the genes involved. aCGH analysis was carried out using a whole-genome 4 × 180 K oligonucleotide microarray platform containing over 170,334 distinct biological probes annotated against NCBI Build 37 (UCSC hg19, February 2009) from Agilent Technologies (5301 Stevens Creek Blvd Santa Clara, CA 95051 United States). Samples from the patient and her parents were hybridized against a same-sex hybridization control (human reference DNA, from Agilent Technologies).

## 3. Results

**Figure 1:**
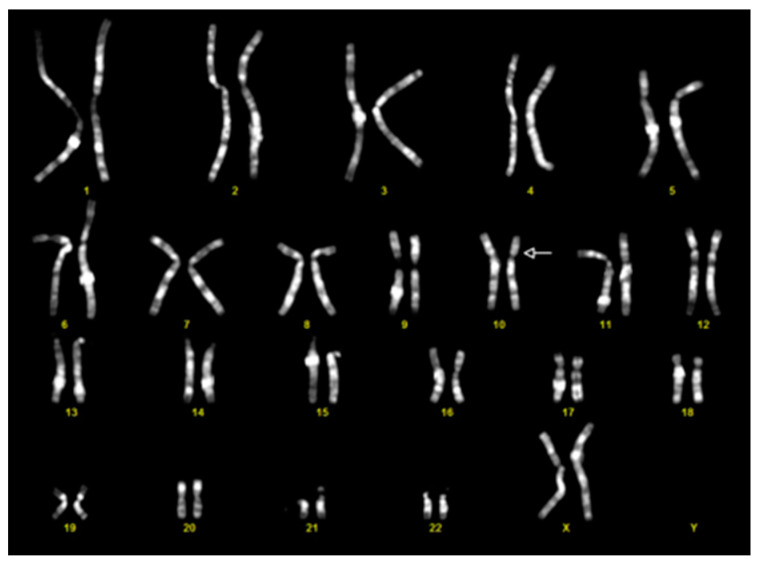
Karyotype of the patient with del(10)(p11.2p11.2). The numbers refer to each couple of homologous chromosomes. The arrow indicates the chromosome involved in the deletion.

Chromosomal Q-banding revealed a karyotype of 46,XX, del(10)(p11.2p11.2) (Figure 1).

**Figure 2:**
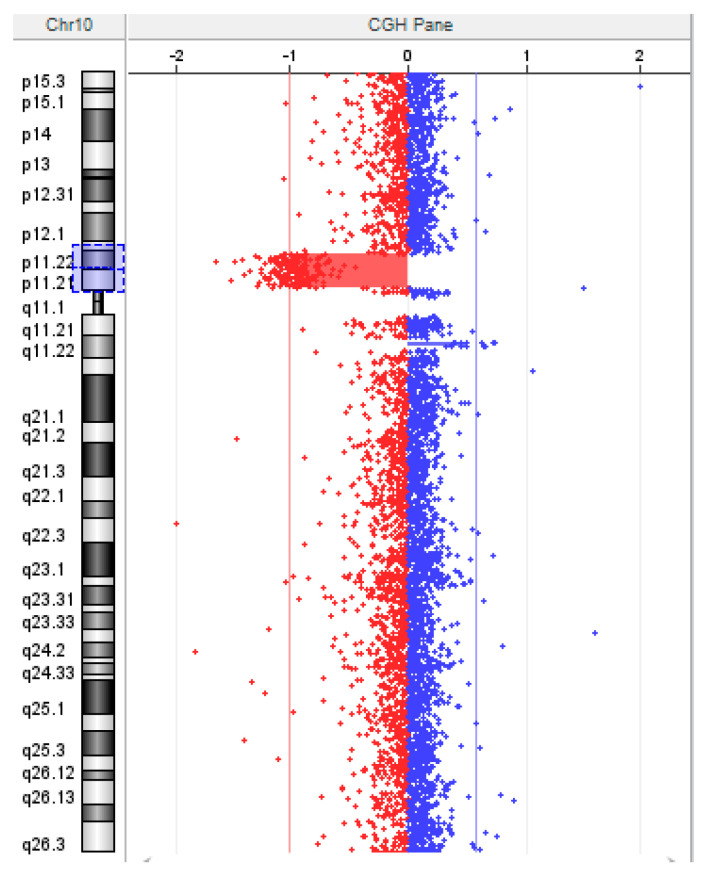
aCGH detected a 5.7 Mb chromosomal deletion in the region of 10p11.21-p11.22 (blue box).

A comparative genomic hybridization (aCGH) array test was performed to establish the breakpoints of the deletion. The CGH array showed the presence of a heterozygous deletion of approximately 5.7 Mb in size at 10p11.21-p11.22: arr[GRCh37]10p11.22p11.21(31615276_37385312)x1 (Figure 2).

This deletion was never classified before; it expands from 10p11.22 to 10p11.21, encompassing 23 genes (Table 1), of which 13 are listed in the OMIM database (Table 1, bold).

Then, we performed aCGH on the DNA extracted from the parents’ peripheral blood. Their normal CMA led us to conclude that the deletion at 10p11.21-p11.22 was de novo.

## 4. Discussion

Here, we report the case of a deletion in a female child aged 5 years old, encompassing 23 genes in the 10p11.2 band. It is known that deletions involving the proximal short arm of chromosome 10 (10p11-p12) are associated with a rare genetic syndromic intellectual disability characterized by developmental delay, hypotonia, speech delay, mild-to-moderate intellectual disability, abnormal behaviour (autistic, aggressive, and hyperactive), and dysmorphic facial features. Congenital heart and brain anomalies, as well as visual and hearing impairment, are also common [1]. In 2011, Wentzel et al. speculated that non-homologous end-joining or FoSTeS might likely be the mechanisms for the recurrence of deletions in this region of chromosome 10, as the breakpoints do not contain any segmental duplications required for NAHR (hon-allelic homologous recombination) [2]. To date, about 13 patients with large overlapping deletions of 10p11.2-p12 have been described in the literature [1,2,3,4,5,6,7]. None of these patients share common breakpoints, but Abdelhedi et al. [5] redefined a new smallest region of overlap (SRO) that contains only the *WAC* gene, thus underlining the importance of this gene in some of the clinical features of these patients, such as intellectual disability and developmental delay. The *WAC* gene encodes the WW-domain-containing adaptor with the coiled-coil region, a nuclear protein that is known to be important in a wide variety of processes, such as gene transcription, microtubule development, autophagy, and Golgi apparatus function [9]. Loss-of-function variants associated with the WAC protein are also associated with DeSanto–Shinawi syndrome, a neurodevelopmental disorder whose clinical features overlap with a large deletion involving this gene [6].

In this study, the chromosomal deletion 10p was found to contain 23 genes, 13 of which are listed in the OMIM database. Interestingly, in this case study, unlike other similar cases reported in the literature, the *WAC* gene, described as the only gene in the SRO region, was not involved in the 10p11-p12 deletion. Although our patient exhibited speech delay, abnormal behaviour (hyperactive), several dysmorphic features, and visual impairment, similar to the majority of patients described in the literature (Table 2), she did not have motor delay. Based on this latest clinical evidence and the absence of the deletion of the *WAC* gene, we speculate that the *WAC* gene could play a role in normal motor development as our patient started to walk independently from the age of 12 months.

**Figure 3:**
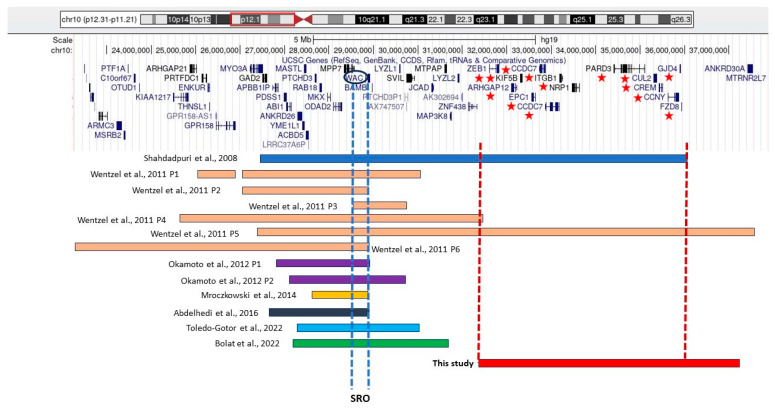
Schematic illustration of chromosome 10 showing the deletions at 10p12p11 in the patients described in the literature (1 patient by Shadadpuri et al., 2008 [1]; 6 patients by Wentzel et al., 2011 [2]; 2 by Okamoto et al., 2012 [3]; 1 by Mroczkowski et al., 2014 [4]; 1 by Abdelhedi et al., 2016 [5]; 1 by Toledo-Gotor et al., 2022 [6]; and 1 by Bolat et al., 2022 [7]) with the patient described in this study. The smallest region of overlap (SRO) identified by Abdelhedi et al. (2016) [5] is indicated by vertical blue dotted lines, while vertical red dotted lines identified patients with overlapping deletion with the patient of this study. The red stars indicated the OMIM genes involved in patient’s deletion.

Furthermore, although the clinical features of the patient described in this study overlapped with those described in the literature, we found that the genes involved were different. In particular, the patient’s deletion overlapped only with three cases described before, but it was the smallest one (Figure 3) and did not share the *WAC* gene associated with the phenotype, as reported in the literature. The patient differed from the other three patients in the absence of developmental delay and cardiac abnormalities. However, both our patient and the three other patients shared visual impairment and some facial dysmorphisms. For example, bulbous nasal tip was observed in patient 4 by Wentzel et al. and in the patient described by Shahdadpuri et al., and a short neck and epichantus were observed in patient 5 by Wentzel et al. These differences and similarities suggest a complex genotype–phenotype correlation, underscoring the importance of thorough clinical assessment and genetic analysis in understanding and managing these conditions.

## 5. Conclusions

In conclusion, we propose that the deletion described here represents a contiguous gene syndrome that is enough to explain the distinct phenotype that partially overlaps with the previous cases reported in the literature, even though different genes are involved. In particular, the *WAC* gene seems to be associated with normal motor development as our patient is the only patient described in the literature with a large deletion in this chromosomal region with the absence of a *WAC* gene deletion and that did not have motor delay. However, it would be useful to further investigate this region to better understand the exact genotype–phenotype correlations.

## Figures and Tables

**Table 1:** The 23 genes involved in the patient deletion: 13 OMIM genes in bold black and the only gene associated with a Mendelian autosomal dominant disease in bold red.

Gene	Protein Name	Protein Function	Gene Ontology
***ZEB1*** **(OMIM #189909)**	Zinc Finger E-Box-Binding Homeobox	The encoded protein seems to contribute to the transcriptional inhibition of interleukin 2. Pathogenic variants in this gene have been linked to late-onset Fuchs endothelial corneal dystrophy and posterior polymorphous corneal dystrophy-3.	*Nucleic acid binding* and *chromatin binding*
*LOC100505502*	POM121 Transmembrane Nucleoporin-Like Protein	This is a pseudogene.	None
***ARHGAP1***	Rho-GTPase-Activating Protein 1	This protein, belonging to a large family, activates enzymes involved in GTP metabolism and may suppress tumour formation by regulating cellular invasion and adhesion.	*GTPase activator activity* and *obsolete SH3/SH2 adaptor activity*
***KIF5B***	Kinesin Family Member 5B	This protein participates in various processes, such as lysosome positioning, natural-killer-cell-mediated cytotoxicity, and the promotion of protein localization to the plasma membrane.	*ATP hydrolysis activity* and *microtubule motor activity*
***EPC1***	Enhancer of Polycomb Homolog 1	This protein works as a transcriptional activator and repressor. It is associated with various processes, like apoptosis, DNA repair, skeletal muscle differentiation, gene silencing, and adult T-cell leukaemia/lymphoma.	*Histone acetyltransferase activity*
*LOC102031319*	Uncharacterized	Uncharacterized.	None
*LOC101929431*	Uncharacterized	Uncharacterized.	None
***CCDC7***	Coiled-Coil Domain-Containing 7	This is a coiled-coil domain-containing protein that is specifically expressed in the testis and may play a role in tumorigenesis.	None
***ITGB1***	Integrin Subunit β 1	This protein plays an important role in cell adhesion and signalling by serving as a receptor for extracellular matrix proteins. It mediates various cellular processes, such as embryogenesis, haemostasis, tissue repair, immune response, and the metastatic diffusion of tumour cells.	*Protein heterodimerization activity* and *signalling receptor binding*
*SNORA86*	Small Nucleolar RNA and H/ACA Box 86	The small nucleolar RNAs, like SNORA86, are involved in the post-transcriptional modification of ribosomal RNAs and gene expression regulation.	None
*IATPR*	ITGB1 Adjacent-Tumour-Promoting LncRNA	This is an RNA gene and is affiliated with the lncRNA class. Diseases associated with IATPR include hepatocellular carcinoma.	None
***NRP1***	Neuropilin 1	This gene encodes one of two neuropilins involved in diverse signalling pathways regulating cell migration, survival, and attraction through interactions with various ligands, such as vascular endothelial growth factor (VEGF) and semaphorins.	*Heparin binding* and *growth factor binding*
*LINC00838*	Long Intergenic Non-Protein-Coding RNA 838	This is an RNA gene and is affiliated with the lncRNA class.	None
***PARD3***	Par-3 Family Cell Polarity Regulator	This gene encodes for a member of the PARD protein family. They affect asymmetrical cell division and direct polarized cell growth.	*Protein phosphatase binding* and *phosphatidylinositol-4,5-bisphosphate binding*
*PARD3-AS1*	PARD3-Divergent Transcript	This is an RNA gene and is affiliated with the lncRNA class.	None
***CUL2***	Cullin 2	A part of Cul2-RING ubiquitin ligase complex. It is predicted to be involved in SCF-dependent proteasomal ubiquitin-dependent protein catabolic processes and protein ubiquitination. It is also predicted to act upstream of, or within, the protein catabolic process.	*Ubiquitin protein ligase binding* and *protein-containing complex binding*
*MIR3611*	MicroRNA 3611	It is an RNA gene and is affiliated with the miRNA class.	None
***CREM***	CAMP-Responsive Element Modulator	This gene codes for a bZIP transcription factor crucial in cAMP-mediated signal transduction during the spermatogenetic cycle and other processes.	*DNA-binding transcription factor activity* and *core promoter sequence-specific DNA binding*
***CCNY***	Cyclin Y	This protein is the CDK14/PFTK1 and CDK16 cyclin-dependent kinase’s positive regulatory subunit that functions as a Wnt signalling pathway cell-cycle regulator.	*Protein kinase binding* and *cyclin-dependent protein serine/threonine kinase regulator activity*
***GJD4***	Gap Junction Protein Delta 4	The encoded protein participates in creating gap junctions, which are intercellular channels that directly link the cytoplasms of contacting cells.	*Not known*
***FZD8***	Frizzled Class Receptor 8	This intronless gene, belonging to the frizzled gene family, encodes a seven-transmembrane domain protein acting as a receptor for wingless-type MMTV integration site family signalling proteins, which are commonly linked to the β-catenin canonical signalling pathway.	*G-protein-coupled receptor activity* and *transmembrane signalling receptor activity*
*MIR4683*	MicroRNA 4683	This is a non-coding RNA RNA gene involved in vitamin-D-dependent rickets (type 2A).	None
*PCAT5*	Prostate-Cancer-Associated Transcript 5	This is a long non-coding RNA. Diseases associated with PCAT5 include prostate cancer and meningeal hemangiopericytoma.	None

**Table 2:** Summary of the clinical features of our case and of previous cases described in the literature affected by the 10p12.1-p11.23 deletion.

	Shahdapuri et al. (2008), [1]	Wentzel et al. (2011), [2]	Okamoto et al. (2012), [3]	Mroczkowski et al. (2014), [4]	Abdelhedi et al. (2016), [5]	Toledo-Gotor et al. (2022), [6]	Bolat et al. (2022), [7]	This Case
**N of cases**	1	6	2	1	1	1	1	1
Gender	M	4F;2M	1F;1M	1M	M	F	M	F
Age at last follow up	1 year	3 to 14 years	6 and 7 years	6 years	3 years	6 years	9 years	5 years
**Genetic**								
Chromosomal band	10p12.1p11.21	10p12.1p11.23 (P1, P2, P3) 10p12.1p11.22 (P4) 10p12.1p11.21 (P5) 10p12.31p11.23 (P6)	10p12.1p11.23	10p12.1p11.23	10p12.1p11.23	10p12.1p11.23	10p12.1p11.23	10p11.22p11.21
Size (Mb)	10	1–10.7	2–2.4	0.85	1.39	2.49	2.8	5.7
Inheritance	De novo	De novo	1 De novo; 1 NR	NR	De novo	NR	NR	De novo
**Dysmorphic facial features**								
Downlslanted palpebral fissures	NR	2/6	2/2	+	+	+	−	−
Synophrys	NR	5/6	2/2	−	+	+	−	−
Deep set eyes	+	4/6	2/2	−	+	+	+	−
Epicanthus	+	NR	2/2	NR	+	NR	−	+
Depressed nasal bridge	NR	1/6	2/2	NR	+	+	−	+
Bulbous nose	+	4/6	1/2	−	+	+	−	+
**Other features**								
Cardiac abnormalities	+	5/6	1/2	−	−	NR	−	−
Motor delay	+	6/6	2/2	+	+	+	+	−
Learning difficulties	+	5/6	1/2	+	+	+	+	+
Hearing impairments	+	2/6	1/2	NR	−	−	NR	−
Visual impairment	+	6/6	NR	+	−	−	+	+
Hyperactivity	NR	4/6	NR	NR	+	+	+	+

Abbreviations: +, feature present; -, feature absent; NR, feature not reported; F, female; M, male; P1, patient1; P2, patient 2; P3, patient 3; P4, patient 4; P5, patient 5; P6, patient 6.

## Data Availability

Raw data were generated in the laboratory of Synlab Italy (Castenedolo, BS, Italy). The data derived supporting the findings of this study are available from the corresponding author upon request.
